# Internally generated conscious contents: interactions between sustained mental imagery and involuntary subvocalizations

**DOI:** 10.3389/fpsyg.2014.01445

**Published:** 2014-12-17

**Authors:** Hyein Cho, Christine A. Godwin, Mark W. Geisler, Ezequiel Morsella

**Affiliations:** ^1^Department of Psychology, San Francisco State UniversitySan Francisco, CA, USA; ^2^School of Psychology, Georgia Institute of TechnologyAtlanta, GA, USA; ^3^Department of Neurology, University of California, San FranciscoSan Francisco, CA, USA

**Keywords:** consciousness, mental imagery, cognitive control, ironic processing, mind wandering, involuntary processing

## Abstract

The conscious field includes not only representations about external stimuli (e.g., percepts), but also conscious contents associated with internal states, such as action-related intentions (e.g., urges). Although understudied, the latter may provide unique insights into the nature of consciousness. To illuminate these phenomena, in a new experimental paradigm [*Reflexive Imagery Task* (RIT)], participants were instructed to not subvocalize the names of visually-presented objects. Each object was presented for 10 s on a screen. Participants indicated whenever they involuntarily subvocalized the object name. Research has revealed that it is difficult to suppress such subvocalizations, which occur on over 80% of the trials. Can the effect survive if one intentionally generates a competing (internally-generated) conscious content? If so, this would suggest that intentional and unintentional contents can co-exist simultaneously in consciousness in interesting ways. To investigate this possibility, in one condition, participants were instructed to reiteratively subvocalize a speech sound (“da, da, da”) throughout the trial. This internally generated content is self-generated and intentional. Involuntary subvocalizations of object names still arose on over 80% of the trials. One could hypothesize that subvocalizations occurred because of the pauses between the intended speech sounds, but this is inconsistent with the observation that comparable results arose even when participants subvocalized a continuous, unbroken hum (“daaa….”) throughout the trial. Regarding inter-content interactions, the continuous hum and object name seem to co-exist simultaneously in consciousness. This intriguing datum requires further investigation. We discuss the implications of this new paradigm for the study of internally-generated conscious contents.

## Introduction

It is a fact of common experience that the conscious field^1^ includes not only representations of external stimuli (e.g., percepts), but also representations of internal states, such as action-related intentions (e.g., urges, Merker, 2013). For example, at one moment in time, one can be conscious of a table, a sign that reads *Coffee*, a song playing in one's head, a coffee mug, and also a strong desire to have a sip of coffee. Most research in experimental psychology and neuroscience has focused on conscious contents associated with perceptuo-semantic processes, especially those that are triggered into existence by external stimuli (see reasons for this in Rosenbaum, 2005; Morsella, 2009). In our example, these kinds of content would be the perception of the table, the mug, and the sign for *Coffee*. However, less investigation has been devoted to illuminating contents associated with internal states such as action-related intentions (the urge to have a sip of coffee) and stimulus-independent mental imagery (a song in one's head). Although understudied, these phenomena may provide unique insights into the nature of consciousness, insights that complement those obtained from more traditional, perception-based research (see Discussion, Morsella and Poehlman, 2013).

To begin to fill this gap in the literature, in an experimental project, we examined the conscious contents associated with action-related intentions (“action-related contents,” for short) and with internally-generated, sustained mental imagery (“sustained imagery,” for short). In addition, we obtained initial data regarding the nature of the interaction between these two kinds of conscious contents (i.e., between action-related contents and sustained imagery). Before discussing these empirical findings and their theoretical implications, it is essential to first describe each kind of conscious content. Thus, first, we describe the nature of, and recent research on, action-related contents; thereafter, we discuss the nature of sustained imagery. These phenomena are of interest to many subfields of psychology and neuroscience, including attention, self-regulation, mental imagery, mind wandering (Smallwood and Schooler, 2006; Mason et al., 2007), and psychopathology (e.g., rumination, Nolen-Hoeksema et al., 2008).

### Internal, action-related contents

Some theorists (e.g., James, 1890; Freud, 1938; Miller, 1959; Vygotsky, 1962; Wegner, 1989) have proposed that action-related contents, though internally-generated, are more predictable and more tied to external control than what might appear to be the case at first glance (see review in Allen et al., 2013). It has been proposed that action-related contents often arise in this predictable and unintentional manner because of the “encapsulated” nature of the generation of conscious content (Fodor, 1983; Krisst et al., in press). Such encapsulation is evident in certain stimulus environments. For example, when one holds one's breath while underwater, or when one runs barefoot across the hot desert sand in order to reach water, one cannot avoid the conscious inclinations to inhale or to avoid touching the hot ground, respectively (Morsella, 2005). The action-related contents triggered by these stimulus environments cannot be weakened or eliminated voluntarily, even when doing so would be adaptive (Öhman and Mineka, 2001; Morsella, 2005). These action-related urges are both externally-triggered and encapsulated from voluntary control. Thus, although inclinations triggered by external stimuli can be *behaviorally suppressible*, they often are not *mentally suppressible* (Bargh and Morsella, 2008).

In line with these conclusions, Helmholtz (1856/1925) concludes that conscious contents can arise from “unconscious inferences” in a manner that resembles the classic, stimulus-response reflex arc. One is conscious of the product (i.e., the conscious content) of these sophisticated and unintentional processes, but not of the processes themselves (Lashley, 1956; Miller, 1962). Helmholtz (1856/1925) notes that such unconscious inferences arise, not only during the generation of basic urges (e.g., for inhaling), but during the generation of action-related processes that are higher-level, as in the case of automatic word reading.

Word reading, though automatic and often effortless, is an unnatural, intellectual process that requires years of extensive training (Helmholtz, 1856/1925). Helmholtz makes the interesting observation that, when an orthographic stimulus (e.g., HOUSE) is presented visually to a subject, the stimulus automatically triggers a conscious representation of the phonological form of the word (i.e., /haus/). Under these quotidian circumstances, the visual stimulus triggers a conscious content that is very different in nature from that of the environmental stimulation that brought the content into existence: The conscious representation is associated, not with vision, but with audition (Levelt, 1989). For object naming, perceptual and conceptual-semantic processing of a stimulus (e.g., the picture of a house) must precede the activation of the phonological form (e.g., /haus/) of the object name (Levelt, 1989). The conscious, phonological form of a word (e.g., /haus/) can be construed as *action-related* because it is considered a “*sub*-vocalized” version of the word (Vygotsky, 1962) and because the nature of the representation is isomorphic to what would be experienced auditorily if the word were uttered aloud (Levelt, 1989).

### The reflexive imagery task and ironic processing

Building on these ideas and on the experimental approaches of Ach (1905/1951), Wegner (1989), and Gollwitzer (1999), Allen et al. (2013) developed a new paradigm, the *Reflexive Imagery Task* (RIT), that allows one to begin to investigate how high-level contents can be activated unintentionally and reliably through external control. In the task, participants are presented with pictures of objects after being instructed to not subvocalize (i.e., name in their minds but not aloud) the names of the objects. To convey the nature of this effect, we will present the reader with a demonstration of the experimental arrangement. Momentarily, we will present you with an object enclosed within parentheses. Your task is to not subvocalize (i.e., “say in one's head”) the name of the object. Here is the stimulus (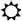). Research demonstrates that the combination of these instructions (which induce a certain *action set*) and the presentation of the external stimulus renders people incapable of suppressing the internally-generated conscious content of the phonological form of the word “sun” (Allen et al., 2013). Did you experience the effect? If so, did it feel as if the word “sun” just popped into consciousness? If it did, then you have experienced what many participants experience during the RIT. When explaining their experience, participants often simply re-describe the effect, “why, of course, if you tell me to not think of the object name, I will.”

Empirical evidence and theorizing suggest that the effect “just happens” to experimental participants and that the effect is not the result of a high-level strategic process on the part of the participant. For example, it appears that the effect does not result from participants consciously thinking in the following manner. “I was instructed to not think of the name of the object. The object is *X*. Therefore, I should not think of *X*.”

Instead, entry into consciousness of the involuntary subvocalization appears to be experienced as immediate and as resulting spontaneously and automatically (Bhangal et al., 2014). This is consistent with theorizing about the nature of this effect. Wegner's (1994) influential model proposes that the unintentional effect arises from the interplay between two distinct mechanisms. One mechanism is an intentional *operating* process that actively searches for mental contents (e.g., sensations and thoughts) capable of maintaining a desired mental state. This process tends to be effortful, capacity limited, and consciously mediated (Wegner, 1994). The other mechanism is an “ironic” *monitoring* process that automatically scans activated mental contents to detect contents signaling the failure to establish the desired mental state. When the monitor detects contents that signify failed control of the operating mechanism, it increases the likelihood that that particular content will enter consciousness, so that the operating mechanism can then process the content and change its operations accordingly. Importantly for present purposes, the ironic monitor mechanism is usually unconscious, autonomous, and requires little mental effort. In most cases of cognitive control, the two mechanisms work together harmoniously. However, harmony fails when the goal in mental control is to not activate a particular mental content (e.g., content *X*), because (a) the operating process can bring only goal-related contents into consciousness and cannot actively exclude contents, and (b) the ironic monitor will reflexively bring into consciousness mental contents (e.g., content *X*) incongruent with the goal at hand. Together, the interaction of the two mechanisms will lead to the automatic activation of content *X* in consciousness. (For treatment of the neural correlates of the two mechanisms, see Wyland et al., 2003; Mitchell et al., 2007. For a review of ironic processing in populations suffering from various psychopathologies, see Magee et al., 2012. For reviews of ironic processing and thought suppression, see Wegner, 1989; Rassin, 2005.)

Despite all the research devoted to ironic processes, the phenomenon of entry of unintentional contents into the conscious field during the RIT is not fully understood (Wegner and Schneider, 2003). The entry event appears to be multifaceted, relying on several component processes (Allen et al., 2013). For example, as outlined in Allen et al. (2013), in order for the experimental effect to arise, there is first the *induction of set* (e.g., to not subvocalize the name of visual objects). Second, this set must be sustained in working memory during the span between the beginning of the trial and the onset of the visual object. During this delay, the action set is thus held in mind, influencing behavioral disposition, but not always influencing consciousness itself. The instructions could therefore be construed as an instance of *imageless thought* (cf., Woodworth, 1915). (Imageless thought was first investigated by theorists of the Würzburg School of Psychology, Schultz and Schultz, 1996.) Last in the process is the presentation of the triggering stimulus (the visual object), which leads to the entry into consciousness of imagery (e.g., subvocalization of the object name). (It is important to note that this punctate, mental event possesses both basic, *phenomenal consciousness*, because it is a conscious experience, and also *access consciousness*, for the conscious content is mentally available for reasoning, rationality, and action; cf., Block, 1995, 2011.)

As an experimental task, the RIT has several positive features. First, the RIT effect is a reliable and easily replicable effect; it occurs in the majority of subjects and in the majority of the experimental trials. For example, in Allen et al. (2013), the effect arose in 86% of the trials (in that study, stimuli were presented for 4 s). Second, the effect is activated unintentionally, via external control, thereby resembling in some ways the nature of reflex action. That the experimental effect is unintentional diminishes the likelihood of experimental artifacts stemming from strategic processing, demand characteristics, or social desirability. (See Allen et al., 2013, p. 1320, for a list of other features that render the RIT a fruitful paradigm for the study of consciousness). Regarding the validity of the measure, evidence from various sources, including neuroimaging and behavioral studies, corroborate that, in paradigms in which participants must report about the experience of a conscious content, it is unlikely that participants confabulate about the occurrence of these mental events (Wyland et al., 2003; Mason et al., 2007; Mitchell et al., 2007; McVay and Kane, 2010). Third, the conscious content associated with the initial version of the RIT (i.e., the phonological form) is well-studied and has well examined properties (Miller, 1996). Interestingly, the conscious content elicited in the RIT is “high-level” because, in terms of stages of processing, the phonological form is post-perceptual and requires complicated transformations, as in the case of object naming, a multi-stage process (Allen et al., 2013).

Fourth, the paradigm also affords one the opportunity to measure, on a trial-by-trial basis, the latency and neural aspects (e.g., as in neuroimaging) of the response processes involved. Fifth, the paradigm is also advantageous in that both stimulus parameters (e.g., word frequency, number of letters, word valence) and instruction (or “action set”) parameters can be varied experimentally. Sixth, the RIT is also beneficial to the field in that it builds nicely on the literature: It builds incrementally on previous research (e.g., Wegner et al., 1987; Gaskell et al., 2001; Smári, 2001), and its effects are predicted by several frameworks (e.g., Miller, 1959; Wegner, 1989; Gollwitzer, 1999; Morsella, 2005). This is the kind of incremental research, involving a robust, multifaceted, and reliable phenomenon that has been investigated for years, that is important for progress in the fields of psychological science and neuroscience (Nosek et al., 2012). Last, the RIT provides a way of examining the mechanisms underlying entry into consciousness, one of the greatest enigmas in science (Crick and Koch, 2003).

As fleshed out below, the primary aim of the present project is to investigate whether the robust and reliable effect of the RIT can arise even when participants intentionally generate a competing (internally-generated) conscious content: sustained imagery. One prediction would be that such competing content would eradicate the RIT effect. If the effect survives such strong interference, the finding would suggest that the RIT effect is even stronger than previous research suggests and that both intentional and unintentional contents can co-exist in consciousness in interesting ways. The intentional, sustained imagery carried out by participants is another form of internally-generated conscious state and our next topic of discussion.

### Sustained imagery

Conscious content, such as sustained imagery, can be held in mind through voluntary mechanisms, such as “top-down” attentional control (Gazzaley and D'Esposito, 2007). In general, executive processes associated with frontal cortex function can sustain the activation of representations in posterior brain regions, regions that have been historically associated with perceptual processing (Enger and Hirsch, 2005; Gazzaley et al., 2005). In this way, during an executive function such as that of “refreshing,” a recently activated representation is reactivated in mind, independently of external, environmental stimulation. Specifically, the process of refreshing entails “the act of thinking of, or foregrounding, a representation of a thought or percept which was activated just a moment earlier and has not yet become inactive” (Johnson and Johnson, 2009, p. 174). Everyday experience and experimental evidence supports the notion that sustaining a representation in mind, voluntarily and for a prolonged span of time, is an effortful process (Farah, 2000). Such a process is often accompanied by *subjective effort* (Robinson and Morsella, 2014).

It is interesting to consider that, during a mental act such as sustaining one representation in mind for a prolonged time, the conscious field is, in a sense, “monopolized” to some extent by that representation. The activation of other conscious contents, if capable of arising simultaneously, must occupy the field along with this content, which is a content that is brought into existence, not by external stimulation or stochastic processes, but by intentional, top-down control. During such feats of intentional, sustained imagery, one may experience, not only the desired content (activated through top-down control), but also spontaneous, task-irrelevant cognitions. In one experiment (Morsella et al., 2010), participants were instructed to perform a concentration exercise in which they had to focus on their breathing for several minutes. During the task, participants experienced and reported task-irrelevant cognitions, including those about a future task that would benefit from forethought. These data exemplify how, when one focuses on the nature of only one conscious content, other, spontaneous contents can still come to mind. These task-irrelevant cognitions can be internally-generated in the sense that they seem unrelated to, and not caused by, stimuli in the external environment. These findings are relevant to research in the fields of “mind wandering” (Smallwood and Schooler, 2006) and rumination (Nolen-Hoeksema et al., 2008).

### The current project

We have reviewed in brief the nature of action-related contents, the RIT, and sustained imagery. The goal of the present experimental project was to further illuminate these phenomena by using the RIT to begin to examine the interactions of internal states in the conscious field. More concretely, our goal was to investigate whether the robust RIT effect could persist even when participants intentionally generate competing conscious contents (i.e., sustained imagery). As mentioned above, one hypothesis would be that such competing content would eradicate the RIT effect. If the effect survives such strong interference, then it would indicate a potential coupling of two kinds of internally-generated states: stimulus-triggered contents and (internally-generated) sustained imagery.

In the present variant of the RIT, participants were instructed to not subvocalize the name of visual objects, such as in the initial version of the task. Each object was presented momentarily (10 s) on a computer screen. Participants indicated by button press each time that they involuntarily subvocalized the object name. As reviewed above, it is difficult for participants to suppress such subvocalizations. In one condition, participants were instructed to reiteratively subvocalize a speech sound (e.g., “da, da, da”) throughout the 10-s trial. This internally generated content was self-generated and intentional. One might hypothesize that subvocalizations could still arise because of the pauses between intended speech sounds; hence, we also investigated whether comparable results arise even when participants subvocalize a continuous, unbroken hum (e.g., “daaa…”) throughout the entirety of the trial. When designing the continuous condition and selecting the intentional imagery, we considered having the sustained imagery be, not a syllable, but an entire word. However, using an entire word introduced too many logistical challenges, including that of ensuring that the intentional imagery of the word (a discrete entity) occur at precisely the same time as the unintentional, stimulus-triggered imagery.

### Inter-content interactions and the possibility of successful suppression of involuntary subvocalizations

This variant of the RIT permits one to begin to examine the nature of inter-content interactions. For example, is it the case that, in the continuous hum condition, the hum and object name could co-exist simultaneously in consciousness? If so, this could reflect that, because of encapsulation, the generation of conscious contents cannot be suppressed (Bargh and Morsella, 2008). Such an intriguing datum would require further investigation. On the other hand, perhaps these two different kinds of internally-generated contents cannot exist simultaneously. It is interesting to consider which contents can, and cannot, exist together in the conscious field. Perhaps if the generation of two different contents relies on the same underlying process, then the two contents cannot exist simultaneously (cf., James, 1890; Cutting, 1976; Navon and Gopher, 1979). This may explain why it seems that one cannot think of two words at the same time (Navon and Gopher, 1979). From this standpoint, the intentional activity on the part of the participants would eradicate the RIT effect. The RIT provides a portal through which to begin to examine such possibilities.

Previous experimental research suggests that, regarding the successful suppression of the involuntary subvocalizations elicited in a task such as the RIT, participants may be using, not only the sustained activation of the subvocalized hum to thwart involuntary subvocalizations, but other strategies as well, including that of self-distraction or “negative cueing” (Wegner et al., 1987, see evidence of successful suppression from self-distraction in Hertel and Calcaterra, 2005). Bulevich et al. (2006), referring to an experimental finding by Hertel and Calcaterra (2005), conclude that “suppression instructions to not think of an unwanted response may succeed if subjects are given the strategy (or themselves hit upon the strategy) of always thinking of some other item when they are trying to suppress an unwanted response” (p. 1575). Voluntarily holding in mind auditory imagery may indeed prevent, or, at least, delay entry of involuntary subvocalizations. In line with this view, in the original RIT study (Allen et al., 2013), participants' responses to the funneled debriefing questions revealed that 8 out of 32 participants attempted to suppress the involuntary subvocalizations by thinking about something else (e.g., subvocalizing a melody) or by subvocalizing about other objects.

Examining whether one conscious content (e.g., an intentional, subvocalized hum) can influence the entry of other contents (e.g., involuntary subvocalizations in the RIT) could yield findings that have implications for the current understanding of, not only cognitive control, consciousness, and mental imagery, but of the basic mechanisms at play during psychopathological processing (e.g., in obsessions, ruminations, intrusive cognitions, compulsions, Nolen-Hoeksema et al., 2008; Magee et al., 2012). Again, our aim was to investigate whether the RIT can be thwarted through competing, sustained imagery. If not, the finding would reveal that both intentional and unintentional contents can co-exist in consciousness in interesting ways.

## Method

### Participants

San Francisco State University students (*n* = 84; 66 females; *M*_Age_ = 24.2 years, *SE* = 0.83) participated for course credit. The involvement of human participants in our project was approved by the Institutional Review Board at San Francisco State University.

### Stimuli and apparatus

Stimuli were presented on an Apple iMac computer monitor (50.8 cm) with a viewing distance of approximately 48 cm. Stimulus presentation and data recording were controlled by PsyScope software (Cohen et al., 1993). Participants used a computer keyboard to indicate their responses to questions and instructions. All questions and instructions were written in black 36-point Chicago font; all fonts and images were displayed on a white background. Participants were presented with a series of 60 well-known objects (Supplementary Material) displayed in the center of the screen, with a subtended visual angle of 6.56° × 5.96° (5.5 × 5 cm). The visual objects were selected on the basis of their high name agreement; these objects were used successfully in previous research (Snodgrass and Vanderwart, 1980; Morsella and Miozzo, 2002; Allen et al., 2013).

### Procedures

In a within-subjects design, each participant experienced each of the three conditions (Baseline [the standard RIT], Continuous Hum, and Punctate Hum). When we initially designed the study, we considered that participants would find it overly challenging to carry out either of the hum conditions (which involve dual-tasking) without first being familiarized with the nature of the RIT. Thus, we initially designed the study so that participants would experience the Baseline condition before experiencing the more multifaceted, subvocalized hum conditions: Following the Baseline condition, half of the participants would experience the Continuous Hum condition, and the other half would experience the Punctate Hum condition. However, for the sake of thoroughness and to diminish any potential order effects from having the Baseline condition presented first, we had half of the participants perform the Baseline condition last. As explained below, the same pattern of results was obtained regardless of whether the Baseline condition was presented first or last. The blocks of trials involving subvocalized humming were always contiguous: Half of the participants first completed the Continuous Hum condition, in which participants were instructed to not think of the name of the object presented while they maintained subvocalization of the hum (e.g., “daaa… ”) for the duration of each trial. The other half of the participants first completed the Punctate Hum condition, in which they were instructed to do the same task, but using an intermittent form of hum (e.g., “da, da, da”).

For all three conditions, instructions were presented on the computer screen, which informed participants that they would be shown a series of objects. Participants were shown each object only once. The objects comprising each list were presented in random order. Presentation order of the three stimulus lists (Supplementary Material) was fully counterbalanced across participants. Upon presentation of the visual object, participants were instructed to press the spacebar as soon as (a) they thought of the name of the object and (b) whenever, thereafter, they thought of the name of object during the rest of the 10 s trial. If participants did not happen to think of the name of the object that was presented, they were instructed to not respond in any way. It was emphasized to participants to press the space bar as quickly as possible as soon as they happened to think of the name of the object. Participants were instructed to keep their eyes focused on the center of the screen at all times during each trial.

**Figure 1:**
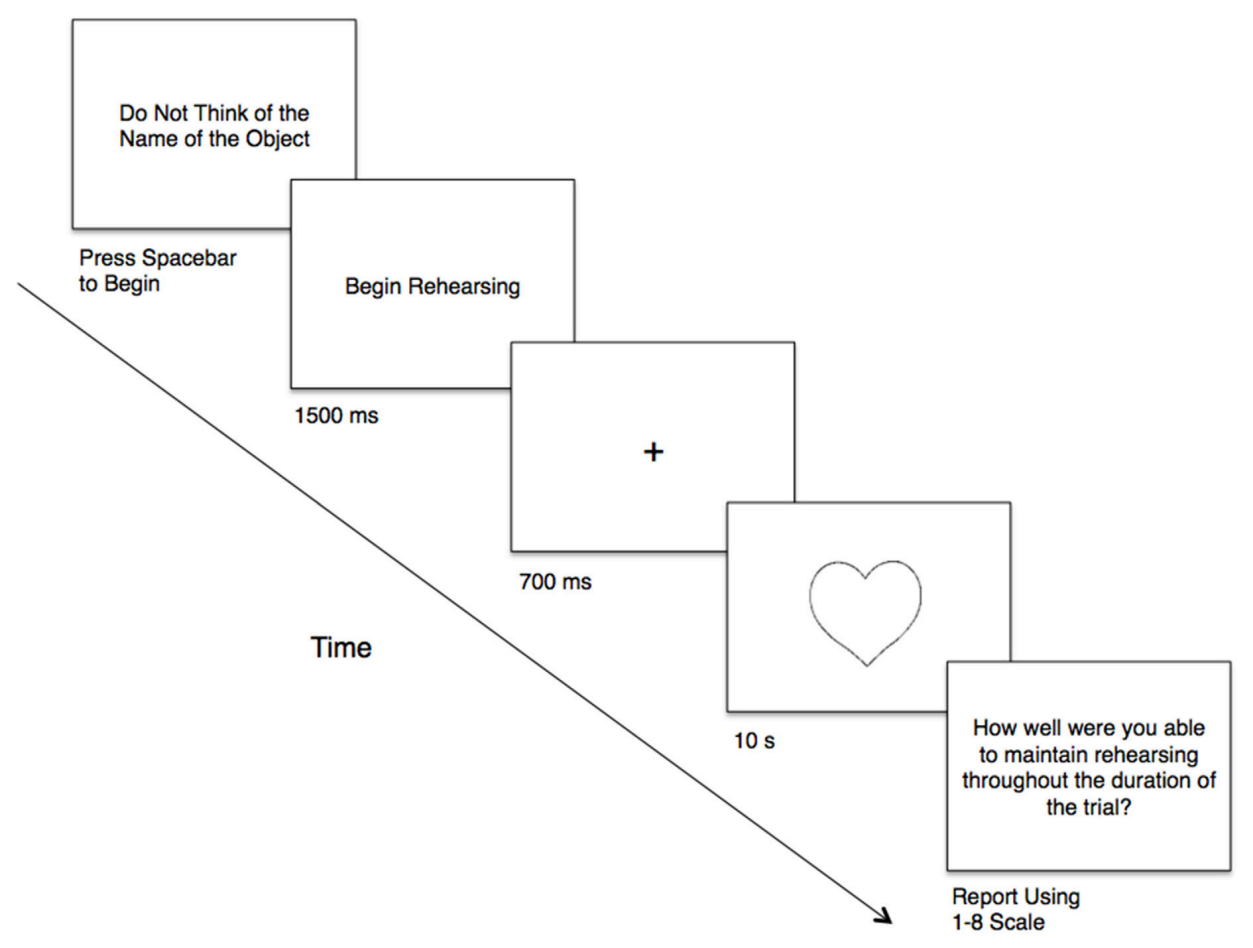
**Schematic depiction of a typical trial**. Not drawn to scale.

Specifically, before each object presentation, the phrase “Do Not Think of the Name of the Object” was displayed in the center of the screen, serving as a ready prompt; participants indicated their readiness by pressing the space bar (Figure 1). For the two humming conditions, a “Begin Rehearsing” prompt appeared on the screen (1,500 ms) following the presentation of “Do Not Think of the Name of the Object.” This prompt allowed participants to initiate the mental rehearsal of the hum before the trial commenced. Once participants indicated their readiness, a fixation-cross (+) appeared in the center of the screen (700 ms), preparing participants for the presentation of the stimulus. Following the fixation, an object appeared for 10 s, during which time participants could indicate, by pressing the space bar, if they happened to think of the name of the object, and for each time that object-naming occurred for the duration of each trial.

Prior to beginning the humming conditions, participants were informed that mental rehearsing is an act of holding something in mind, such as holding in mind something that one has to remember. For example, if one has to remember a phone number while looking for a pen, one often rehearses the phone number mentally until one can write it down. Participants were told that this is called mental rehearsal. Participants were instructed that they would perform a form of mental rehearsal during each trial of the humming conditions. Upon receiving the instructions to perform mental rehearsal, participants were informed about the distinction between continuous and punctate subvocalized humming. Participants practiced how to carry out subvocalized humming for each condition by imitating the instructor, who spoke the hum aloud (e.g., “da, da, da,” for the Punctate Hum condition and “daaa…” for the Continuous Hum condition). Participants were then asked to repeat the hum, but silently (subvocalized).

Following this training, in the Continuous Hum condition (trials = 20), participants were instructed to mentally sustain a steady, uninterrupted form of hum throughout the duration of each trial. For the Punctate Hum condition (trials = 20), participants were instructed to mentally repeat a discrete, intermittent, or “piece-by-piece” form of hum throughout the duration of each trial. For all three conditions, participants first completed a practice trial to familiarize themselves with the task and to assure the experimenter that they were following the instructions. At the end of the practice trial, participants were verbally asked whether they had any questions regarding the task. The stimuli used during the practice trials were held constant across participants and were distinct from the stimuli used during the experimental trials.

For both humming conditions, participants were reminded to start mentally rehearsing with a prompt “Begin Rehearsing” before they were presented with an object. We took the time to collect some additional data. Following each of the humming trials, participants were asked: “*How well were you able to maintain rehearsing throughout the duration of the trial?*” Participants indicated their response to the question using a one-to-eight continuous scale, with 1 signifying “not well at all” and 8 signifying “very well.” Throughout the experimental session, the experimenter verified that participants were following instructions.

At the conclusion of the experiment, participants responded to a series of funneled debriefing questions (following the procedures of Bargh and Chartrand, 2000), which included general questions to assess whether (a) participants were aware of the purpose of the study, (b) participants had any strategies for completing the task, (c) anything interfered with performance on the task, and (d) participants felt that they tried, or intended, to follow instructions. We also asked participants whether or not they had a strategy when attempting to not think of the name of the object. Because the study included participants who were non-native speakers of English, we also included a series of questions to assess whether (e) participants thought of the name of the object in a language other than English, (f) participants pressed the space bar in such a situation, and (g) participants had a strategy for completing the task if they happened to think of the name of the object in more than one language. Importantly, participants were also asked questions regarding their ability to carry out the mental rehearsal, to assess whether (h) participants were able to maintain mental rehearsal for each kind of subvocalized humming, and (i) one form of rehearsal was more effective than the other when trying to not think of the name of the visual object. From 84 participants, data from 76 participants were included in the analysis. The data for 8 participants were excluded from analyses because (a) participants did not follow instructions (e.g., looking away from the screen when stimuli were presented) or (b) equipment malfunction (e.g., unexpected shut-down of the computer software).

## Results

### Primary dependent measure: proportion of trials with unintentional subvocalizations

Importantly, the RIT effect survived the strong, sustained imagery manipulation: As was found in previous experiments using the RIT, unintentional subvocalizations occurred (at least once) on a substantial proportion of the 20 trials comprising each of the three conditions (*M*_Baseline_ = 0.87, *SE* = 0.02; *M*_Continuous Hum_ = 0.83, *SE* = 0.03; *M*_Punctate Hum_ = 0.83, *SE* = 0.03). For each of the three conditions, the mean rate was significantly different from zero, *t*s > 26.38, *p*s < 0.0001.

An ANOVA with the three levels of Baseline, Continuous Hum, and Punctate Hum yielded a main effect of condition, *F*_(2, 150)_ = 3.66, *p* = 0.028, η^2^_*p*_ = 0.05. However, the same analysis with arcsine transformations of the proportion data revealed that the effect of condition is actually non-significant, *F*_(2, 150)_ = 2.05, *p* = 0.133, η^2^_*p*_ = 0.03. (Arcsine transformations are often used to statistically normalize data that are in the form of proportions.) We can conclude that, for all conditions, involuntary subvocalizing occurred frequently and at comparable rates, on over 80% of the trials. That these involuntary subvocalizations occurred even during the Continuous Hum condition suggests that the sustained imagery and involuntary subvocalizations co-existed in the conscious field.

As mentioned above, the Baseline condition was counterbalanced across participants such that, for our final sample of 76 participants, it appeared as the first and last block an equal number of times (i.e., 38 times). Nevertheless, we examined whether order of presentation of this block of trials influenced our primary dependent measure. In a mixed ANOVA with Baseline Order as a between-subjects factor and Condition (Baseline, Continuous Hum, and Punctate Hum) as a within-subjects factor, there was no main effect of Baseline Order, *F*_(1, 74)_ = 0.01, *p* = 0.91, a main effect of condition, *F*_(2, 148)_ = 3.67, *p* = 0.028, and no interaction between the two factors, *F*_(2, 148)_ = 1.14, *p* = 0.322. This additional analysis provides further evidence that our primary effect of condition was not an artifact of order of presentation of the Baseline condition. Similar null effects (*F*s < 1, *p*s > 0.60) of Baseline order are found in an ANOVA with arcsine transformations of the proportions, except that, in this case, the effect of condition was non-significant, *F*_(2, 148)_ = 2.03, *p* = 0.135.

### Subvocalizations per trial

One of our secondary dependent measures was the number of instances of unintentional subvocalizing per trial. This dependent measure was comparable across the three conditions, with the per-condition means spanning from 1.53 to 1.88 (range = 0.35; Table 1). Importantly, for each of the three conditions, the mean number of instances was significantly different from zero, *t*s > 13.43, *p*s < 0.0001.

**Table 1:** **Mean number of occurrences of unintentional subvocalizations per trial and mean latencies (ms) of first subvocalization as a function of condition**.

**BASELINE**		
Mean number	1.88	(*SD* = 1.22, *SE* = 0.14, range = 0.05–6.15)
Latency	2323.91	(*SD* = 1183.01, *SE* = 135.70)
**PUNCTATE SUBVOCALIZED HUMMING**		
Mean number	1.64	(*SD* = 0.98, *SE* = 0.11, range = 0–5.45)
Latency	2501.00	(*SD* = 1050.41, *SE* = 122.11)
**CONTINUOUS SUBVOCALIZED HUMMING**		
Mean number	1.53	(*SD* = 0.77, *SE* = 0.09, range = 0.05–3.65)
Latency	2415.92	(*SD* = 997.34, *SE* = 114.40)

The duration of each trial was 10 s.

**Figure 2:**
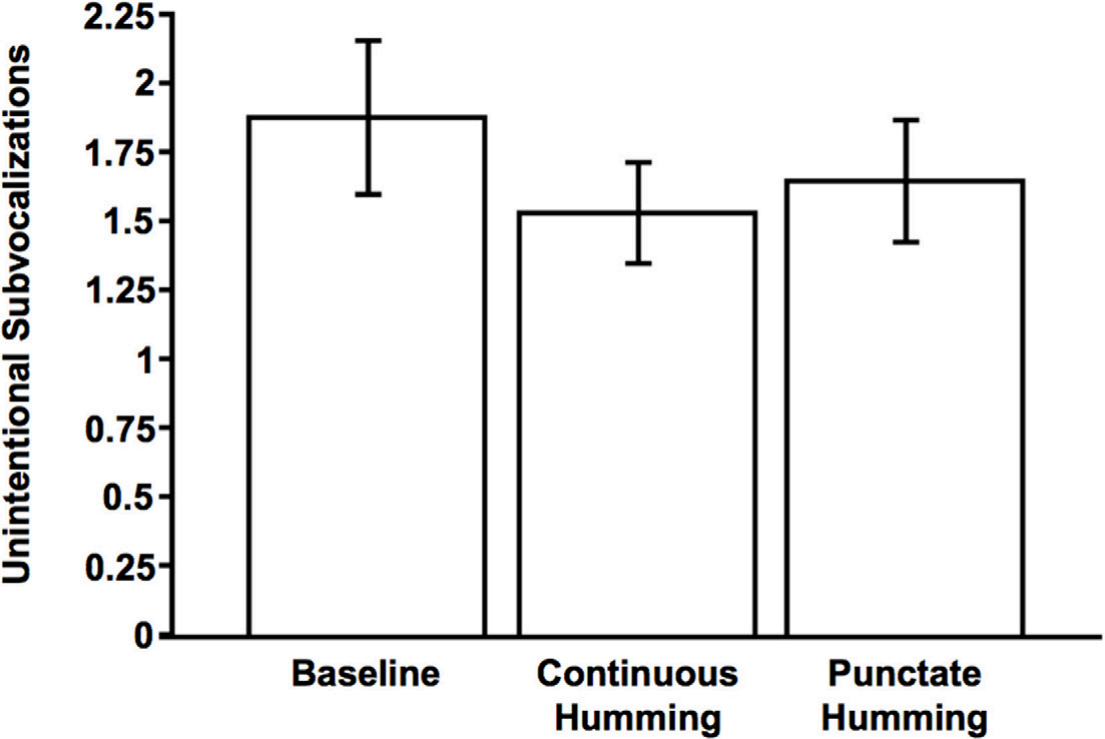
**Mean number of unintentional subvocalizations as a function of condition (Baseline, Continuous Hum, Punctate Hum)**. Error bars indicate 95% confidence interval.

An ANOVA with three levels (Baseline, Continuous Hum, and Punctate Hum) revealed a significant main effect of condition on this measure, *F*_(2, 150)_ = 9.43, *p* < 0.001, η^2^_*p*_ = 0.11 (Figure 2). Planned contrasts revealed the Baseline condition was significantly different from either of the humming conditions, *t*s > 2.80, *p*s < 0.01. This pattern of results provides corroboratory evidence that participants were in fact performing the sustained imagery. However, the two humming conditions led to comparable effects, *t*_(75)_ = 1.70, *p* = 0.094.

We examined whether order of presentation of Baseline block of trials influenced our measure. In a mixed ANOVA with Baseline Order as a between-subjects factor and Condition (Baseline, Continuous Hum, and Punctate Hum) as a within-subjects factor, there was no main effect of Baseline Order, *F*_(1, 74)_ = 1.65, *p* = 0.204, η^2^_*p*_ = 0.02, a main effect of condition, *F*_(2, 148)_ = 9.38, *p* < 0.001, η^2^_*p*_ = 0.11, and no interaction between the two factors, *F*_(2, 148)_ = 0.64, *p* = 0.528, η^2^_*p*_ = 0.01. This additional analysis provides further evidence that our effect of condition was not an artifact of the order of presentation of the Baseline condition.

### Latency

We examined the mean latency of the first experienced subvocalization following the beginning of a trial (Table 1). An ANOVA revealed that condition had a main effect on this measure, *F*_(2, 146)_ = 4.40, *p* = 0.014, η^2^_*p*_ = 0.06. However, this main effect was driven only by the difference between Baseline and Punctate, *t*_(73)_ = 2.87, *p* = 0.005; the other contrasts were non-significant, *t*s < 1.08, *p*s > 0.28.

### Trial-by-trial perceived difficulty of subvocalized humming

The self-report ratings (1–8 scale) regarding the difficulty of maintaining the intentional imagery throughout the trial (i.e., in response to the question, “*How well were you able to maintain rehearsing throughout the duration of the trial?*”) revealed that, as one would expect (and consistent with Robinson and Morsella, 2014), it was easier for participants to sustain, throughout the trial, the punctate imagery (*M* = 5.86, *SE* = 0.16) than the continuous imagery (*M* = 5.65, *SE* = 0.16), *t*_(75)_ = 2.34, *p* = 0.022. This difference provides further corroboration that participants were following instructions and performing, as instructed, the two different kinds of imagery.

## Discussion

In our project, we used a new experimental paradigm to investigate the nature of two internally generated states. Specifically, we examined both externally-elicited conscious content and internally-generated, sustained imagery. In addition, we obtained some initial data regarding the nature of the interaction between these two kinds of conscious contents. Below, we discuss the implications of these new findings. Because much remains unknown regarding these states and about this new paradigm, our conclusions are conservative and tentative.

Our paradigm affords one the ability to examine further, not only self-generated states, but an intriguing and reliable phenomenon: ironic processing. We do not pretend to provide a complete account of this interesting and robust phenomenon (cf., Wegner and Schneider, 2003). Our findings replicate and extend previous findings regarding ironic processing (see reviews of findings in Wegner, 1989; Rassin, 2005). The present research is incremental, robust, and involves a multifaceted phenomenon that, we believe, could illuminate much about the unconscious processes operating over supraliminal stimuli (cf., Bargh and Morsella, 2008). As mentioned above—and as may have been experienced by the reader in our “sun” demonstration above—the entry into consciousness of the unintentional subvocalization appears to be immediate and not due to high-level, strategic processing. Consistent with this conclusion, on some trials, the latencies of the unintentional subvocalization appeared too short to reflect strategic processing. In addition, such strategic processing would have been difficult for our participants to carry out during the interference conditions. The notion that the RIT effect is not caused by experimental demand or high-level strategic processes is also consistent with the extant process model of the phenomenon (Wegner, 1994), in which activation of the undesired content in consciousness is the automatic aspect of ironic processing. It is also consistent with the recent observation that the subvocalizations are influenced systematically by factors such as word frequency (Bhangal et al., 2014). The latter finding is unlikely to result from experimental demand or strategic processing. For example, regarding the former, such an artifact would require for participants to have a theory regarding how word frequency should influence responses in an experiment.

**Figure 3:**
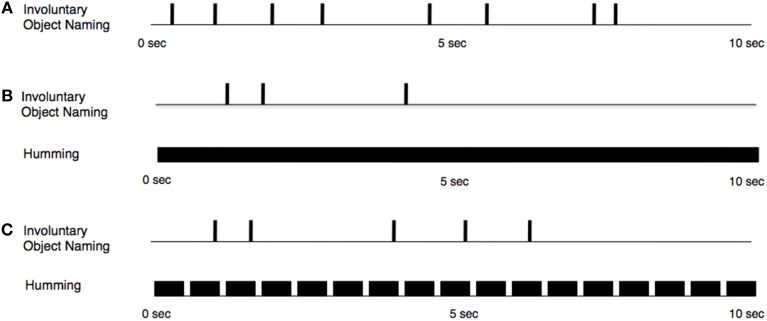
**Schematic representation of the temporal properties of conscious, intentional subvocalized humming (B, Continuous Hum; C, Punctate Hum) and unintentional subvocalizations throughout a hypothetical trial:** Vertical lines indicate points in time at which participants reported stimulus-triggered, unintentional subvocalizations. In the Baseline condition **(A)**, participants were not instructed to subvocalize any form of humming.

Our RIT effect arose despite our interference manipulations, which, in one case, included a continuous and unbroken subvocalized hum (Figure 3). Moreover, the unintentional effect was observed on roughly the same percentage of trials (over 80%) as had been observed in experiments lacking any interference (e.g., Allen et al., 2013). The same conclusion can be drawn from the analysis of the rates of unintentional subvocalizations across trials or from the analysis of the mean number of unintentional subvocalizations per trial: the RIT effect was robust and, for both dependent measures and across the three conditions, the effect was always significantly different from zero. The latency of the first unintentional subvocalization tended to be shorter for the Baseline condition than for the Punctate condition. This observation corroborates what participants explicitly reported to the experimenter. Moreover, the difference found in the trial-by-trial ratings regarding the difficulty of sustaining the imagery (in which participants reported that it was easier to sustain the punctate imagery than the continuous imagery) provides further corroboration that participants were following instructions and instantiating, as instructed, the two different kinds of imagery. Perhaps our interference manipulation would have thwarted the RIT effect if another kind of imagery (e.g., complete words) had been sustained in working memory. As mentioned above, there are logistical challenges encountered when attempting to use whole words as the form of sustained imagery in trials spanning several seconds.

Nevertheless, in an investigation of the occurrence of internally-generated, conscious thoughts, one cannot avoid the technique of self-report, which brings with it well-known limitations. For example, self-reports can be inaccurate, as when memory of fleeting mental contents leads to incorrect self-reports (Block, 2007). In addition, participants may base their responses on heuristics or strategies regarding how one should comport oneself in an experiment (see Discussion in Morsella et al., 2009). Given the striking robustness and reliability of the RIT phenomenon (as perhaps experienced by the reader in response to our demonstration above), we do not believe that these well-known limitations undermine the validity of our primary findings.

Regarding instances of successful suppression, until more data are obtained, we remain agnostic regarding whether participants' performance (e.g., as reflected in their latencies) is consistent with “inhibition” accounts of cognitive control (cf., Aron, 2007; Levy and Wagner, 2011) or with other accounts, such as Jamesian ideomotor approaches in which successful suppression is interpreted as resulting, not from direct inhibition of the undesired action plan, but from the sustained activation of an incompatible action plan (see Hommel, 2009).

Our finding that the stimulus-triggered subvocalization arose despite participants' intentions, and despite the fact that the conscious field was occupied by other contents (e.g., the sustained imagery), is consistent with theorizing about the encapsulated nature of the generation of conscious contents (Fodor, 1983; Krisst et al., in press). From the standpoint of Krisst et al. (in press), this encapsulation is built into the system because it would be maladaptive for the generation of conscious contents to be controlled completely by one's beliefs or desires (see also Pylyshyn, 1984; Firestone and Scholl, 2014). From this standpoint, and consistent with the notion of the unconscious inference (Helmholtz, 1856/1925), the RIT effect reflects the nature in which most conscious contents are (and should be) generated—automatically and independently of one's volition. Contents reflecting intentional, top-down processing are a small subset of all conscious contents. Our finding is also consistent with approaches that regard conscious contents as “action options” that, though activated in the conscious field, need not influence action directly (Allen et al., 2013). (Investigators have begun to examine the behavioral consequences of such unselected action options, Filevich and Haggard, 2013.) Together, these views concerning “action options” and about the encapsulated nature of content generation may have implications for our understanding of the basic mechanisms in psychopathological phenomena (e.g., in obsessions, ruminations, intrusive cognitions, compulsions, Nolen-Hoeksema et al., 2008; Magee et al., 2012).

Building on Filevich and Haggard (2013), future investigations could focus on the behavioral consequences of the unintentional subvocalizations triggered by the RIT. In addition, research could examine whether participants perceive the sustained imagery as associated with “the self” and perceive the unintentional imagery as “foreign to the self” (cf., Riddle and Morsella, 2009; Montemayor et al., 2013). It is our hope that future studies will build on this paradigm and on our findings, thereby yielding more insights about these elusive, self-generated states.

### Conflict of interest statement

The authors declare that the research was conducted in the absence of any commercial or financial relationships that could be construed as a potential conflict of interest.

## Footnotes

^1^The conscious field comprises of everything that one is conscious of at one time. We are here referring to the most basic form of consciousness, such as the experience of the color red or of an urge to breathe when holding one's breath. This form of consciousness has perhaps been best defined by the philosopher Nagel (1974), who proposed that an organism possesses consciousness if there is something it is like to be that organism—something it is like, for example, to be human and experience pain, love, or breathlessness. Similarly, Block (1995) claimed, “the phenomenally conscious aspect of a state is what it is like to be in that state” (p. 227).
