# Supplementary material for: Internally generated conscious contents: interactions between sustained mental imagery and involuntary subvocalizations
Source: Front Psychol. 2014 Dec 17;5:1445. doi: 10.3389/fpsyg.2014.01445 (PMC4269111; doi:10.3389/fpsyg.2014.01445)
Supplement: Supplementary file 1 [file DataSheet1.DOCX]

| **Appendix** | | | |
| --- | --- | --- | --- |
| List of the visual objects (line drawings) | | |  |
| Anchor | Flower | Pipe |  |
| Apple | Fork | Plug |  |
| Ball | Glasses | Radio |  |
| Balloon | Guitar | Rake |  |
| Banana | Gun | Ring |  |
| Bear | Hammer | Ruler |  |
| Bed | Hand | Saw |  |
| Bicycle | Heart | Scissors |  |
| Book | Horse | Shoe |  |
| Cake | House | Star |  |
| Candle | Igloo | Stool |  |
| Cat | Iron | Television |  |
| Chair | Key | Tie |  |
| Couch | Ladder | Traffic Light |  |
| Crown | Lamp | Tree |  |
| Dog | Mouse | Trophy |  |
| Door | Mouth | Umbrella |  |
| Envelope | Nose | Wheel |  |
| Fire | Pan | Whistle |  |
| Flag | Pencil | Windmill |  |
